# Case Report: Cholangioscopic and histopathological findings before and after treatment in immune-related cholangitis

**DOI:** 10.3389/fimmu.2026.1934941

**Published:** 2026-10-05

**Authors:** Yu Akazawa, Takuto Nosaka, Shouichi Namikawa, Arisa Saito, Yosuke Murata, Tomoko Tanaka, Kazuto Takahashi, Tatsushi Naito, Masahiro Ohtani, Yasunari Nakamoto

**Affiliations:** 1 Department of Gastroenterology, Faculty of Medical Sciences, University of Fukui, Fukui, Japan; 2 Graduate School of Health and Human Life Sciences, Fukui Prefectural University, Fukui, Japan

**Keywords:** bile duct biopsy, cholangioscopy, immune checkpoint inhibitor, immune-related cholangitis, treatment response

## Abstract

Immune-related cholangitis is a rare immune-related adverse event, and its diagnostic criteria and treatment strategies have not yet been established. Here, we report a case of immune-related cholangitis in which treatment response could be objectively visualized through reassessment of cholangioscopic and histopathological findings before and after treatment. A 79-year-old man with recurrent cutaneous squamous cell carcinoma of the scalp developed high fever and general fatigue after six courses of nivolumab, and laboratory tests revealed marked elevation of hepatobiliary enzymes. Contrast-enhanced computed tomography and endoscopic ultrasonography showed bile duct wall thickening, endoscopic retrograde cholangiopancreatography demonstrated shaggy irregularity in the distal bile duct, and peroral cholangioscopy revealed diffuse surface irregularity and granular changes of the biliary mucosa. Furthermore, cholangioscopy-guided bile duct biopsy demonstrated marked lymphocytic infiltration, particularly infiltration of CD8-positive T cells, leading to a diagnosis of immune-related cholangitis. Prednisolone and ursodeoxycholic acid were initiated, with nivolumab discontinued, after which the clinical symptoms and hepatobiliary enzyme levels gradually improved. Furthermore, on Day 28 after treatment initiation, reassessment by cholangioscopy and bile duct biopsy confirmed improvement in the cholangioscopic findings and reduction of lymphocytic infiltration, particularly CD8-positive T-cell infiltration, on bile duct biopsy. These objective findings supported tapering of steroid therapy. To our knowledge, very few cases have described reassessment of cholangioscopic and histopathological findings before and after treatment in immune-related cholangitis. We suggest that such evaluation may contribute to confirming treatment response and guiding therapeutic strategy in immune-related cholangitis.

## 1 Introduction

Immune checkpoint inhibitors (ICIs) have markedly improved outcomes in various cancer types and are becoming established as a standard treatment for most solid tumors (1). While ICIs have potent antitumor effects, they can also induce immune-related adverse events (irAEs), with grade 3/4 irAEs occurring in approximately 10%–15% of patients (2–4). ICI-induced irAEs most commonly affect the gastrointestinal tract, endocrine organs, skin, and liver (2). Although the mechanisms responsible for irAEs have not been fully elucidated, several potential mechanisms have been proposed, including enhanced T-cell activity against antigens shared by tumor and normal tissues, increased levels of preexisting autoantibodies, and elevated inflammatory cytokines (2).

Immune-related cholangitis is relatively rare, with an incidence of approximately 0.05-4.5% (5, 6). In addition, immune-related cholangitis is often refractory to treatment and can occasionally be life-threatening, underscoring the importance of early diagnosis and appropriate therapeutic intervention. However, established diagnostic criteria and treatment strategies remain unclear. Immune-related cholangitis mainly presents with abdominal pain and jaundice, and radiologic and endoscopic examinations typically show bile duct dilatation, bile duct wall thickening, and bile duct irregularity (5). In addition, cholangioscopy is useful because it allows direct visualization of the biliary mucosa, enabling more precise evaluation of these lesions (7). Also, histopathological evaluation by bile duct biopsy is indispensable for excluding other diseases and establishing a definitive diagnosis (8). Furthermore, reassessment of cholangioscopic and histopathological findings before and after treatment may be important for establishing treatment protocols, including steroid tapering.

We present a case of immune-related cholangitis in which cholangioscopic and histopathological findings were evaluated before and after treatment, allowing clear observation of treatment-related changes. To our knowledge, very few reports have described assessment of these findings before and after treatment. This case is meaningful in that it allowed objective visualization of the treatment response in immune-related cholangitis.

## 2 Case description

A 79-year-old man was diagnosed with cutaneous squamous cell carcinoma of the scalp (T4aN0M0, stage IVA) and underwent curative surgical resection for the lesion. He had no prior history of systemic anticancer therapy, including immune checkpoint inhibitors, and received no adjuvant systemic therapy after surgery. Local recurrence at the same site on the scalp was observed 4 months after surgery, and nivolumab monotherapy was administered at a dose of 240 mg every 2 weeks for a total of six courses. Four days after the final administration, he developed a high fever and general fatigue, which persisted for 1 week, leading to hospitalization at our institution. No adverse events had occurred during the preceding nivolumab treatment.

On admission, laboratory findings revealed elevated hepatobiliary enzyme levels (AST 62 U/L, ALT 39 U/L, total bilirubin 0.8 mg/dL, γ-glutamyl transpeptidase 1709 U/L, and alkaline phosphatase 767 U/L) and an elevated inflammatory response (white blood cell count 4600/mm³ and C-reactive protein 3.55 mg/dL) (**Supplementary Table 1**). Antinuclear and antimitochondrial antibodies were negative, and the serum IgG4 level was 17.4 mg/dL. Hepatitis B surface antigen and hepatitis C virus antibody were negative, and both blood and bile cultures were negative. No new medications other than nivolumab had been initiated, and no changes had been made to the patient’s concomitant medications before the onset of hepatobiliary abnormalities. The patient had no known history of pre-existing biliary disease. Contrast-enhanced computed tomography showed heterogeneous hepatic enhancement in the early phase, with mild wall thickening of the bile duct and gallbladder (**Figures 1A, B**), but showed no gallstones or common bile duct stones and no evidence of a malignant obstructive process, including tumor recurrence causing biliary obstruction. Endoscopic ultrasonography also revealed thickening of the common bile duct wall and gallbladder wall (**Figures 1C, D**). Furthermore, endoscopic retrograde cholangiopancreatography showed marked shaggy irregularity in the distal bile duct (**Figures 2A, B**), and mild pruned-tree appearance was also observed in the peripheral bile ducts. Peroral cholangioscopy showed diffuse surface irregularity of the bile duct mucosa, with granular changes particularly prominent in the distal bile duct (**Figure 2C**). Biopsy specimens obtained from the distal bile duct showed marked lymphocytic infiltration, particularly of CD8-positive T cells (**Figures 3A, B**). Similar findings were also observed in biopsy specimens obtained from the hilar region and the common bile duct near the cystic duct junction. Based on these endoscopic and pathological findings, the patient was diagnosed with immune-related cholangitis. Also, according to the characteristic cholangiographic abnormalities, diffuse bile duct wall thickening, and cholangioscopic findings, the present case was classified as large duct-type immune-related cholangitis. According to the Common Terminology Criteria for Adverse Events (CTCAE) version 5.0 criterion for “alkaline phosphatase increased, “ the hepatobiliary toxicity was classified as Grade 4. The ALP level at the initiation of nivolumab was 78 U/L (institutional reference range, 38–113 U/L), and the peak ALP level reached 2300 U/L (20.4 × the upper limit of normal). The peak laboratory values are summarized in **Supplementary Table 1**. Seven days after admission, the inflammatory response and hepatobiliary enzyme levels worsened further. Because the patient showed progressive deterioration despite conservative management, prednisolone was initiated at 50 mg/day (approximately 1 mg/kg/day) together with oral ursodeoxycholic acid 900 mg/day for the treatment of Grade 4 immune-related cholangitis. After the initiation of treatment, the inflammatory response and hepatobiliary enzyme levels gradually improved (**Supplementary Table 1**), and the fever and fatigue resolved.

**Figure 1:**
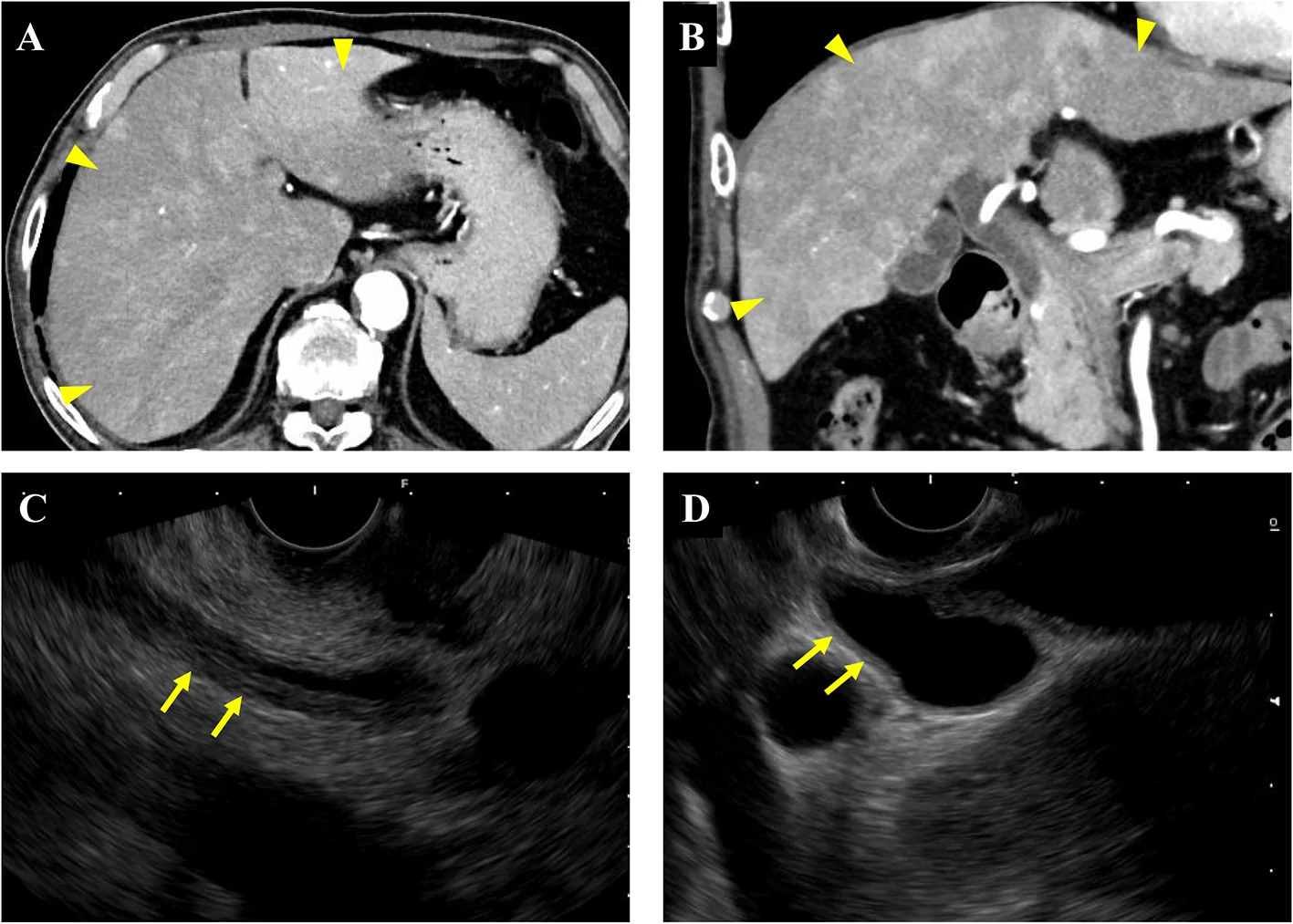
Initial computed tomography and endoscopic ultrasound findings suggestive of immune-related cholangitis. **(A, B)** Axial and coronal contrast-enhanced computed tomography images showing heterogeneous hepatic enhancement in the liver parenchyma (yellow arrowheads). **(C)** Endoscopic ultrasound image demonstrating thickening of the common bile duct wall (yellow arrows). **(D)** Endoscopic ultrasound image demonstrating thickening of the gallbladder wall (yellow arrows).

**Figure 2:**
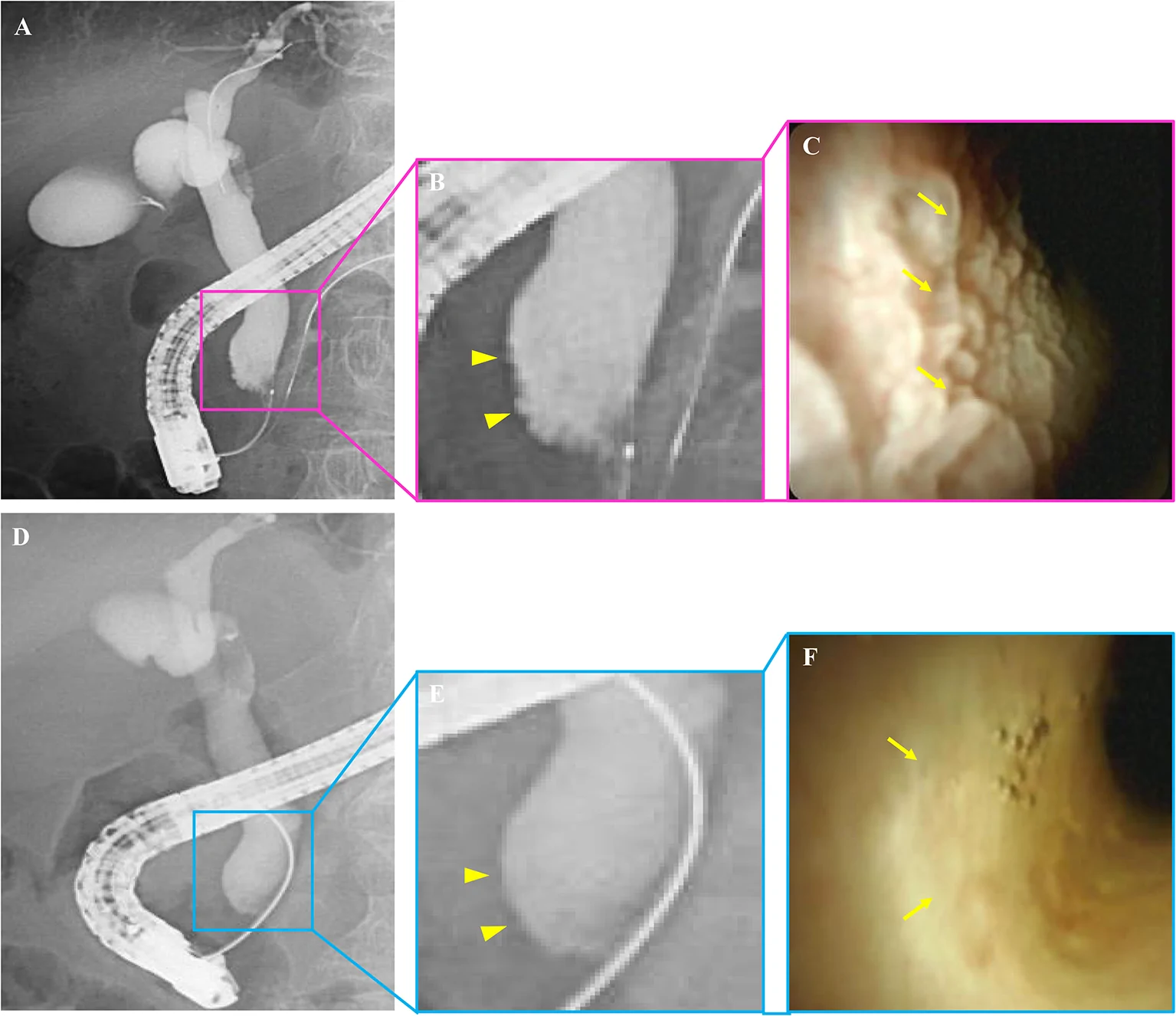
ERCP fluoroscopic images and cholangioscopic findings before and after treatment. The findings shown are representative images obtained from the same distal bile duct segment before and after treatment. Image interpretation was independently performed by two experienced pancreatobiliary endoscopists, both of whom confirmed improvement in the mucosal findings following treatment. **(A)** ERCP fluoroscopic image before treatment. **(B)** Magnified fluoroscopic view of the affected bile duct segment before treatment, showing shaggy irregularity. **(C)** Pre-treatment cholangioscopic image demonstrating diffuse surface irregularity and granular change of the biliary mucosa (arrows). **(D)** ERCP fluoroscopic image after treatment. **(E)** Magnified fluoroscopic view of the previously affected segment after treatment, showing improvement of the shaggy irregularity. **(F)** Post-treatment cholangioscopic image demonstrating improvement of the diffuse surface irregularity and granular mucosal change, with scar formation (arrows).

**Figure 3:**
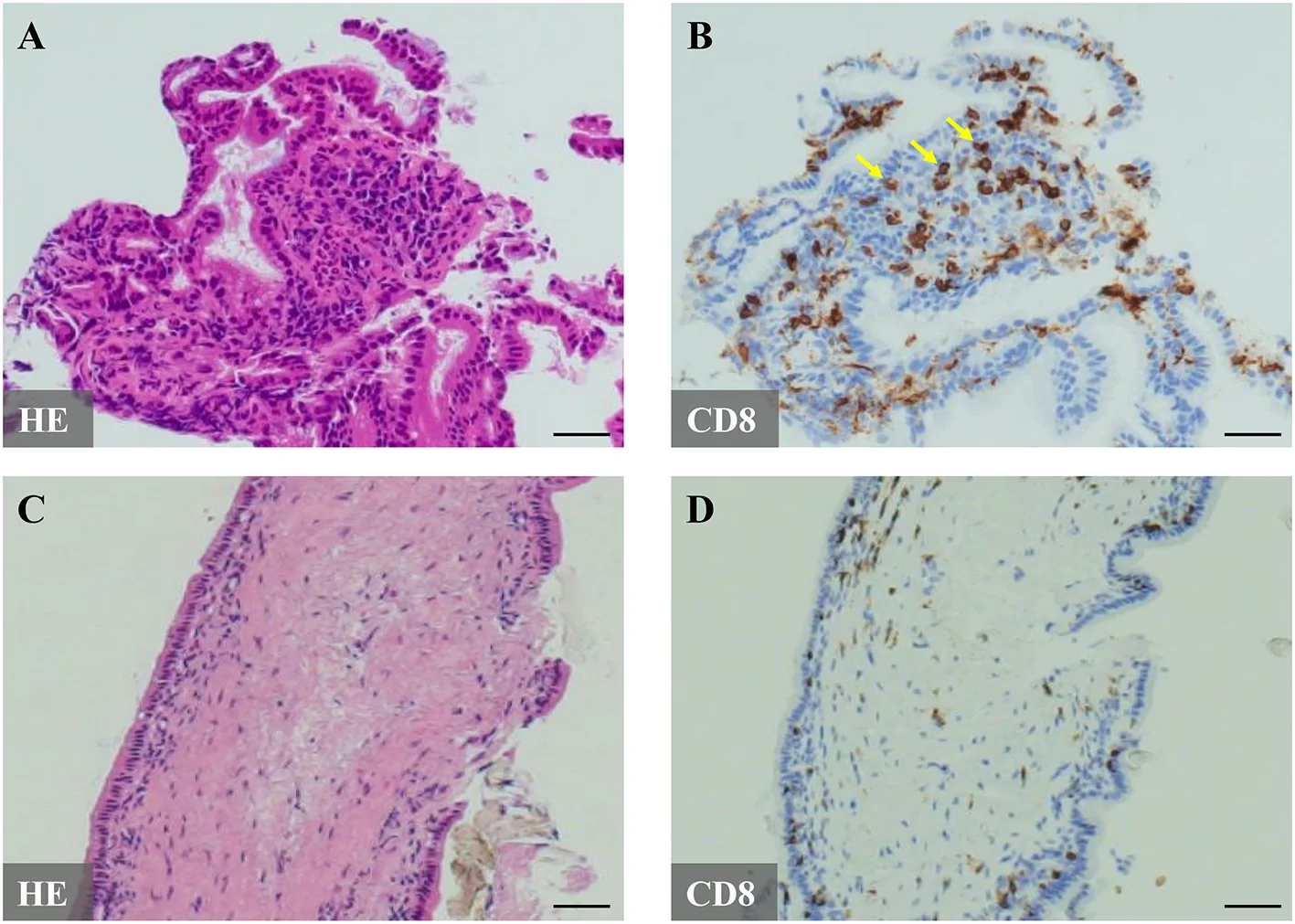
Histopathological findings before and after treatment. Representative images from three biopsy specimens obtained from the same distal bile duct segment before and after treatment under direct cholangioscopic guidance are shown. Semiquantitative pathological assessment of all biopsy specimens demonstrated that CD8-positive inflammatory cell infiltration decreased from severe before treatment to mild after treatment. **(A)** Hematoxylin and eosin staining of the pre-treatment bile duct specimen showing lymphocytic infiltration. **(B)** CD8 immunostaining of the pre-treatment specimen showing infiltration of CD8-positive T cells (arrows). **(C)** Hematoxylin and eosin staining of the post-treatment specimen showing reduced lymphocytic infiltration. **(D)** CD8 immunostaining of the post-treatment specimen showing reduced infiltration of CD8-positive T cells. The specimens were routinely stained with hematoxylin and eosin. CD8 immunohistochemistry was performed using a monoclonal antibody against CD8 (clone 4B11, ready-to-use; Leica Biosystems) on a Leica BOND-III system (Leica Biosystems), with heat-induced epitope retrieval using BOND Epitope Retrieval Solution 2 and detection using the BOND Polymer Refine Detection system. Scale bars: 100 μm.

By Day 28, the patient’s symptoms and hepatobiliary enzyme levels had improved, and the prednisolone dose had been tapered to 20 mg/day. However, non-contrast CT still showed mild residual gallbladder wall thickening. In addition, persistent or recurrent immune-related cholangitis has been reported. Therefore, further evaluation was considered clinically warranted to assess residual biliary inflammation and to guide subsequent steroid tapering. Accordingly, endoscopic retrograde cholangiopancreatography, cholangioscopy, and bile duct biopsy were performed again on Day 28. Before the repeat procedures, the clinical rationale and purpose of the procedures, as well as their invasive nature, were explained to the patient, and written informed consent was obtained. No procedure-related complications occurred. Specifically, endoscopic retrograde cholangiopancreatography showed improvement of the shaggy irregularity in the distal bile duct that had been observed before treatment (**Figures 2D, E**). In addition, cholangioscopy demonstrated improvement in the surface irregularity and granular changes of the bile duct mucosa, with scar formation (**Figure 2F**). Cholangioscopic examinations before and after treatment were performed in the same distal bile duct segment to ensure consistency of evaluation. The cholangioscopic images were independently reviewed by two experienced pancreatobiliary endoscopists, both of whom confirmed improvement in the mucosal findings after treatment. Bile duct biopsy revealed decreased lymphocytic infiltration, particularly decreased infiltration of CD8-positive T cells (**Figures 3C, D**). Biopsy specimens were obtained from the same distal bile duct segment under direct cholangioscopic guidance before and after treatment to ensure sampling consistency. Three biopsy specimens were obtained at each time point. Because of the small size of the biopsy specimens, no predefined number of high-power fields or formal validated scoring system was used; instead, the entire evaluable tissue in each specimen was reviewed. The same two pathologists, who were aware of the timing of the biopsies, evaluated the specimens and comprehensively categorized the degree of lymphocytic and CD8-positive inflammatory cell infiltration into three levels (mild, moderate, or severe). Both lymphocytic and CD8-positive inflammatory cell infiltration were categorized as severe before treatment and mild after treatment, with decreased infiltration consistently observed in all three post-treatment biopsy specimens. Subsequently, contrast-enhanced computed tomography performed on Day 56 after the initiation of treatment showed improvement of the heterogeneous hepatic enhancement in the early phase that had been observed before treatment. Furthermore, hepatobiliary enzyme levels continued to improve on laboratory testing. Based on these findings, immune-related cholangitis was considered to have improved, and steroid therapy was gradually tapered and maintained at 5 mg/day (**Supplementary Table 1**). Thereafter, the patient remained free of relapse and was discharged on Day 65. The overall clinical course is summarized in **Figure 4**. After discharge, the patient continued to be followed at our institution. Prednisolone 5 mg/day and ursodeoxycholic acid 900 mg/day were continued, and no recurrence of immune-related cholangitis or steroid-related adverse events was observed during follow-up at our institution. Approximately 3 months after discharge, because of deterioration in activities of daily living and general condition, the patient was referred to a hospital closer to his home for best supportive care. Nivolumab rechallenge was not attempted. The patient died approximately 6 months after discharge because of progression of the primary disease.

**Figure 4:**
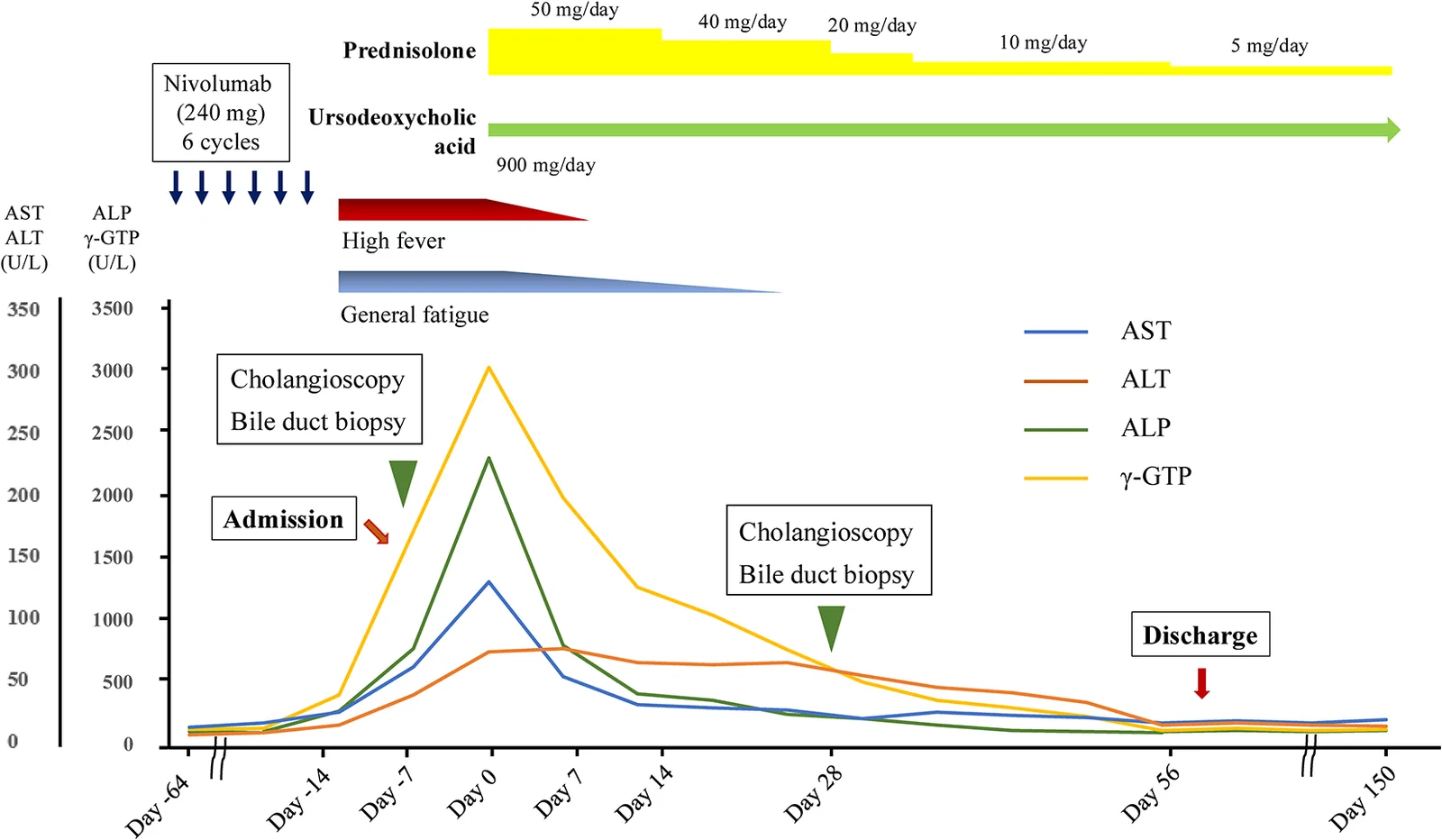
Clinical course of the patient. Clinical timeline showing nivolumab administration, onset of symptoms, laboratory progression, diagnostic investigations, initiation of prednisolone and ursodeoxycholic acid, repeat ERCP, cholangioscopy, and bile duct biopsy, tapering of prednisolone, and hospital discharge. After discharge, the patient continued to be followed at our institution and was referred to another hospital approximately 3 months after discharge for best supportive care. The patient subsequently died approximately 6 months after discharge because of progression of the primary disease.

## 3 Discussion

Among immune checkpoint inhibitor-related adverse events, immune-related cholangitis is very rare, with an incidence of 0.05–4.5% (5, 6); however, it can occasionally become life-threatening. Clinical symptoms of immune-related cholangitis include abdominal pain or discomfort (48%), fever (21%), malaise (12%), and jaundice (10%) (9–11), and blood tests often reveal liver injury with marked elevation of biliary enzymes such as γ-glutamyl transpeptidase and alkaline phosphatase (12–14). In addition, Kawakami et al. reported that radiologic features of immune-related cholangitis include extrahepatic bile duct dilatation without obstruction and diffuse thickening of the extrahepatic bile duct wall (15). These findings were also consistent with those in our case. Also, Onoyama et al. showed that the median number of ICI administrations before onset of immune-related cholangitis was 5.5 (range, 1–27) (5). In our case, immune-related cholangitis also developed after six courses of nivolumab, which was generally consistent with the previous report. However, diagnostic criteria and treatment strategies for immune-related cholangitis have not yet been established. Diagnosis requires the exclusion of common bile duct stones, malignancy, and complications, as well as differentiation from IgG4-related sclerosing cholangitis and primary biliary cholangitis (15). In the present case, infectious cholangitis, malignant biliary obstruction, viral hepatitis, IgG4-related sclerosing cholangitis, other drug-induced liver injury, and pre-existing biliary disorders were carefully excluded based on laboratory findings, cross-sectional imaging, ERCP, cholangioscopy, and histopathological examination; a diagnosis of immune-related cholangitis was made. Therefore, in patients receiving ICIs, immune-related cholangitis should always be kept in mind to allow early detection and early therapeutic intervention.

Cholangioscopy is extremely useful because it allows direct visualization of the biliary mucosa and thereby contributes to detailed evaluation of mucosal lesions in immune-related cholangitis. Previous reports have shown various cholangioscopic findings in immune-related cholangitis, including ulceration, burned-out epithelium, diverticulum-like findings, membranous stenosis, and haustra-like changes (7, 16–18). In our case, diffuse surface irregularity and granular changes of the biliary mucosa were observed in the common bile duct, and these findings were considered consistent with immune-related cholangitis. In addition, to our knowledge, only six previously reported cases of immune-related cholangitis were evaluated by cholangioscopy (7, 16–18) (**Table 1**). These cases were identified through a PubMed search of case reports of immune-related cholangitis published through April 2026, from which reports involving cholangioscopic evaluation were selected. Although the number of reports is limited, these previous reports also suggest that cholangioscopy is useful for the diagnosis of immune-related cholangitis. Furthermore, histopathologically, marked inflammatory cell infiltration of the bile duct, particularly infiltration of CD8-positive T cells, has been considered a characteristic finding of immune-related cholangitis (5, 11, 19, 20). In our case, cholangioscopy-guided bile duct biopsy also showed marked lymphocytic infiltration, particularly infiltration of CD8-positive T cells. Moreover, as in our case, cholangioscopy-guided bile duct biopsy may be a more useful approach because biopsy can be performed while directly observing the biliary mucosa. Based on these findings, direct mucosal evaluation by cholangioscopy and histopathological evaluation by bile duct biopsy are both extremely important for the diagnosis of immune-related cholangitis.

**Table 1:** Reported cases of immune-related cholangitis evaluated by cholangioscopy.

Author, year	Age/Sex ^a^	Primary malignancy	Symptoms	Time to onset	Imaging findings (EUS/CT/ERCP)	Cholangioscopic findings	Pathology	Treatment	Post-treatment evaluation in cholangioscopy and pathology	Outcome ^b^
Takeda, 2022 (7)	70/F	Lung Cancer	No symptoms	N/A	• Localized bile duct wall thickening • Irregularity of the bile duct wall	• Diffuse erythema of the bile duct wall	• Infiltration of lymphocytes	PSL	• Decrease in the haustra-like change in the bile duct • Ongoing infiltration of lymphocytes	Alive
Onoyama, 2019 (16)	61/M	Bladder Cancer	Fever	4 months	• Localized bile duct wall thickening • Irregularity of the bile duct wall	• Band-like bile duct stricture • Diverticulum-like outpouching	• Infiltration of inflammatory cells	UDCA PSL	None	Alive
Onoyama, 2019 (16)	89/M	Bladder Cancer	No symptoms	3 months	• Localized bile duct wall thickening • Irregularity of the bile duct wall	• Band-like bile duct stricture	• Infiltration of inflammatory cells	UDCA	None	Alive
Onoyama, 2019 (16)	63/M	Lung Cancer	No symptoms	5 months	• Localized bile duct wall thickening • Irregularity of the bile duct wall	• Band-like bile duct stricture • Diverticulum-like outpouching	• Infiltration of inflammatory cells	UDCA	None	Alive
Kuraoka, 2018 (17)	69/M	Lung Cancer	No symptoms	3 months	• Localized bile duct dilatation • Irregularity of the bile duct wall	• Multiple ulcers with easy bleeding	• Infiltration of inflammatory cells and lymphocytes	PSL	None	Died
Tomishima, 2022 (18)	68/F	Lung Cancer	Fever	2 months	• Diffuse bile duct wall thickening • Feathery appearance of the bile duct	• Edematous bile duct wall	• Infiltration of inflammatory cells	PSL	None	Alive
Our case	79/M	Skin Cancer	Fever	5 months	• Heterogeneous hepatic enhancement • Diffuse bile duct wall thickening • Feathery appearance of the bile duct	• Diffuse surface irregularity of the bile duct • Granular biliary mucosa	• Infiltration of CD8- positive T cells	UDCA PSL	• Improved mucosal abnormalities with scar formation • Reduced CD8-positive T cell infiltration	Alive

^a^ Sex. M, male; F, female.

^b^ Outcome was defined as the clinical outcome of immune-related cholangitis rather than all-cause survival. In the present case, the patient subsequently died because of progression of the primary disease, without recurrence of immune-related cholangitis during follow-up at our institution.

CT, Computed Tomography; ERCP, Endoscopic Retrograde Cholangiopancreatography; EUS, Endoscopic Ultrasonography; N/A, not applicable; PSL, prednisolone; UDCA, ursodeoxycholic acid.

To our knowledge, only a few reports have serially evaluated cholangioscopic and histopathological findings before and after treatment in immune-related cholangitis (**Table 1**). Takeda et al. reported that, in a patient with immune-related cholangitis, cholangioscopic findings improved 3 months after steroid therapy, whereas lymphocytic infiltration remained present (7). In our case, after treatment, improvement in cholangioscopic findings, including surface irregularity and granular changes, as well as reduction of lymphocytic infiltration, particularly CD8-positive T-cell infiltration, on bile duct biopsy, were confirmed. Therefore, this case is meaningful in that it enabled objective visualization of the treatment response for immune-related cholangitis through cholangioscopic and histopathological reassessment.

Steroid therapy is the first-line treatment for immune-related cholangitis, similar to that for other immune-related adverse events (6, 11, 21–23). However, only 11.5–14% of patients reportedly achieve normalization of hepatobiliary enzymes with steroid therapy alone, suggesting that the response to steroids is often limited (5, 15, 24–28). In addition, recurrence is also observed in some cases (27, 28), and no clear consensus has yet been reached regarding the appropriate intensity of steroid therapy, tapering speed, or the introduction of additional treatment. In our case, after the initiation of treatment, both the clinical symptoms and hepatobiliary enzyme levels improved promptly. Subsequently, further improvement in the cholangioscopic and histopathological findings was confirmed, and steroid therapy could be tapered based on these objective findings. The contribution of these objective findings to therapeutic decision-making is one of the important clinical implications of this case. Although marked clinical, endoscopic, and histopathological improvement was observed after treatment initiation, these improvements cannot be attributed solely to prednisolone. Ursodeoxycholic acid was initiated concurrently, and nivolumab had already been discontinued before steroid therapy. Therefore, the favorable clinical course observed in the present case may have resulted from the combined effects of corticosteroid therapy, concomitant ursodeoxycholic acid administration, and discontinuation of nivolumab. In addition, in steroid-refractory or recurrent cases, serial evaluation of cholangioscopic and histopathological findings may also be important for more accurately assessing treatment response and determining therapeutic strategy. In the future, we suggest that optimal methods for treatment evaluation and therapeutic strategies in immune-related cholangitis may need to be established through further accumulation of irAE cholangitis cases.

This case report has several limitations. First, this was a single-case report, and therefore the findings cannot be generalized. Second, the cholangioscopic assessment was primarily qualitative, and biopsy findings may have been influenced by sampling variability despite careful sampling from the same biliary segment. In addition, the possibility that the histological improvement observed on repeat biopsy partly reflected local healing after the initial biopsy cannot be excluded. Although decreased lymphocytic infiltration was consistently observed in all three biopsy specimens obtained at the repeat examination, the contribution of biopsy-related healing cannot be completely distinguished from the treatment-associated changes. Third, because prednisolone and ursodeoxycholic acid were initiated concurrently, the independent contribution of corticosteroid therapy to the observed improvement could not be determined. Finally, repeat ERCP, cholangioscopy, and bile duct biopsy are invasive procedures. Therefore, repeat invasive assessment should be considered only in carefully selected patients when objective evaluation of treatment response is clinically warranted, rather than as a routine approach.

## 4 Conclusion

In conclusion, we experienced a rare case of immune-related cholangitis in which treatment response could be objectively visualized through reassessment of cholangioscopic and histopathological findings before and after treatment. Such evaluation may contribute to confirming treatment response and guiding therapeutic strategy in immune-related cholangitis.

## 5 Patient perspective

Because the patient had dementia and his general condition and performance status deteriorated due to progression of the underlying malignancy, an accurate patient perspective could not be obtained. Throughout the clinical course, the treatment strategy, including the rationale, potential benefits, and risks of the diagnostic and therapeutic procedures, was thoroughly discussed with his eldest son and other family members. They demonstrated understanding of the patient’s clinical condition and agreed with the treatment strategy through shared decision-making.

## Data Availability

The raw data supporting the conclusions of this article will be made available by the authors, without undue reservation.
