# Supplementary material for: Case Report: Cholangioscopic and histopathological findings before and after treatment in immune-related cholangitis
Source: Front Immunol. 2026 Oct 5;17:1934941. doi: 10.3389/fimmu.2026.1934941 (PMC13677885; doi:10.3389/fimmu.2026.1934941)
Supplement: Supplementary file 2 [file Table1.docx]

Supplementary Material

# 1 Supplementary Table

| Supplementary Table 1: Serial laboratory findings before and after treatment. | | | | | | | | | |
| --- | --- | --- | --- | --- | --- | --- | --- | --- | --- |
| Day | Day -7 ^♰ *^ | Day -4 | Day 0 ^#^ | Day 4 | Day 7 | Day 14 | Day 28 ^*^ | Day 35 | Day 56 |
| Prednisolone dose (mg/day) | - | - | 50 | 50 | 50 | 40 | 20 | 10 | 5 |
| WBC (/µL) | 4600 | 6700 | 8400 | 6600 | 7100 | 9000 | 6900 | 9100 | 7700 |
| CRP (mg/dL) | **3.55** | **8.20** | **9.20** | **2.52** | 0.97 | **1.06** | 0.80 | **1.24** | 0.89 |
| T-Bil (mg/dL) | 0.8 | 1.0 | **1.7**^**^ | 0.7 | 0.6 | 0.7 | 0.5 | 0.4 | 0.4 |
| AST (U/L) | **62** | **95** | **130**^**^ | **68** | **54** | 31 | 27 | 20 | 21 |
| ALT (U/L) | 39 | **60** | **74**^**^ | **64** | **76** | **65** | **65** | **55** | 33 |
| γ-GTP (U/L) | **1709** | **2495** | **3023**^**^ | **2386** | **1979** | **1257** | **759** | **498** | **221** |
| ALP (U/L) | **767** | **1634** | **2300**^**^ | **1406** | **788** | **403** | **236** | **200** | 98 |
| ^♰^ Day -7 corresponds to the day of admission.  ^#^ Day 0 was defined as the day steroid therapy was initiated.  ^*^ ERCP, cholangioscopy, and bile duct biopsy were performed on Day -7 and Day 28.  ^**^ The values shown represent the peak laboratory values observed during the clinical course. The institutional reference ranges were as follows: T-Bil, 0.4–1.5 mg/dL; AST, 0–30 U/L; ALT, 0–30 U/L; γ-GTP, 0–50 U/L; and ALP, 38–113 U/L.  Abbreviations: ALP, alkaline phosphatase; ALT, alanine aminotransferase; AST, aspartate aminotransferase; CRP, C-reactive protein; γ-GTP, γ-glutamyl transpeptidase; T-Bil, total bilirubin; WBC, white blood cell count. | | | | | | | | | |
